# The AhR ligand phthiocol and vitamin K analogs as *Pseudomonas aeruginosa* quorum sensing inhibitors

**DOI:** 10.3389/fmicb.2022.896687

**Published:** 2022-09-14

**Authors:** Tianyuan Jia, Dongjing Liu, Xianbiao Bi, Menglu Li, Zhao Cai, Jiapeng Fu, Zhi Liu, Pengyao Wu, Xue Ke, Aiqun Jia, Guoliang Zhang, Guobao Li, Liang Yang

**Affiliations:** ^1^School of Medicine, Southern University of Science and Technology, Shenzhen, China; ^2^Shenzhen Third People's Hospital, National Clinical Research Center for Infectious Disease, The Second Affiliated Hospital of Southern University of Science and Technology, Shenzhen, China; ^3^School of Pharmaceutical Sciences, Hainan University, Haikou, China; ^4^Shenzhen Key Laboratory of Gene Regulation and Systems Biology, Southern University of Science and Technology, Shenzhen, China

**Keywords:** aryl hydrocarbon receptor, phthiocol, vitamin K, *Pseudomonas aeruginosa*, *Pseudomonas* quinolone signal

## Abstract

The aryl hydrocarbon receptor (AhR) protein senses microbial-secreted metabolites to trigger the host's innate immune system. The *Pseudomonas* quinolone signal (PQS) and *Mycobacterium tuberculosis* (MTb) metabolite phthiocol (Pht) are both ligands of AhR with similar chemical structures. As PQS is an essential quorum-sensing molecule that regulates a wide range of virulence factors in *Pseudomonas aeruginosa*, we hypothesized that Pht and its analogs are potential *P. aeruginosa* quorum-sensing inhibitors (QSIs) with immune-modulating functions. In this study, we demonstrated that Pht was able to inhibit the *P. aeruginosa pqs* QS system and reduce both biofilm formation and the production of pyocyanin. Molecular docking analysis suggested that Pht competes with PQS at the binding site of its receptor, PqsR. An electrophoretic mobility shift assay confirmed the Pht-PqsR interaction and showed that Pht attenuated PqsR from binding to the *pqsA* promoter. Proteomic analysis showed that synthesis of the key *pqs* QS proteins decreased upon the addition of Pht to the bacterial cultures. Furthermore, Pht analogs vitamins K_1_ (Phylloquinone), K_2_ (Menaquinones), and K_3_ (Menadione) were also showed to inhibit the *P. aeruginosa pqs* QS system while able to activate the AhR signaling pathways. Our study suggests that the AhR ligands Pht and its vitamin K analogs are promising QSIs for the alternative treatment of *P. aeruginosa* infections.

## Introduction

The aryl hydrocarbon receptor (AhR) protein is a highly conserved ligand-dependent transcription factor that senses xenobiotics to activate detoxifying monooxygenase cytochrome P4501 (CYP1) for degrading ligands to metabolites; it also plays a key role in immune control (Leclerc et al., 2021). Besides its well-known ligands-environmental pollutants, AhR can also sense secreted microbial metabolites such as pigments and signaling molecules. Recent studies showed that AhR can recognize the *Pseudomonas aeruginosa* quorum-sensing molecule 2-heptyl-3-hydroxy-4 (1H)-quinolone (*Pseudomonas* quinolone signal, PQS) (Moura-Alves et al., 2019) and *M. tuberculosis* (MTb) metabolite 2-hydroxy-3-methyl-1,4-naphthoquinone (phthiocol, Pht) (Moura-Alves et al., 2014) as its ligands. After binding, AhR either activates or suppresses the expression of its regulated genes that are involved in a wide range of pathways, such as cell signaling, innate immune response, and the control of inflammation levels against bacterial infections. Since PQS and Pht are both ligands of AhR with similar chemical structures, we hypothesized that these two molecules may be competitive in binding the native PQS receptor in *P. aeruginosa*, PqsR. A very early study reported that Pht inhibits the growth of *Streptococcus pyogenes, Escherichia coli*, and other bacteria (Lichstein and Van De Sand, 1946). Recently, Pht was shown to exhibit moderate anti-*P.aeruginosa* activity due to its metal ion chelating capacity (Shinde and Wadekar, 2018), which is also a feature of the PQS molecule. However, it remains unclear whether Pht is indeed able to interfere with *P. aeruginosa* PQS signaling.

*Pseudomonas aeruginosa* is a widespread opportunistic gram-negative bacterium responsible for many human infections (Curran et al., 2018). The chronic infection caused by multi-drug resistant *P. aeruginosa* is the leading cause of death in patients with cystic fibrosis (Holmes et al., 2021). *Mycobacteria* (such as MTb and *Mycobacterium abscessus*) and *P. aeruginosa* both cause diseases in human lungs and share the same ecological niche (Ehrt et al., 2018). Recent studies suggest that these two pathogens are likely to cause co-infection and aggravate inflammatory lung disease (Falkinham et al., 2015). Clinical studies on tuberculosis with recurrent *P. aeruginosa* infection in the lower respiratory tract showed that these patients have refractory sepsis and low immunity (Dos Santos et al., 2012). Moreover, the incidence of *P. aeruginosa* infection in patients with tuberculosis is very high, significantly increasing the risk of in-hospital death among patients with pulmonary TB (Martiniano et al., 2014). As MTb and *P. aeruginosa* share the same ecological niche in the human body, they may compete for limited nutrients in the lung (Devi et al., 2021). Thus, we hypothesized that there may be some mechanism of agonism between these two species.

Several strategies to combat *P. aeruginosa* biofilm infections have been developed in recent years, including compounds that do not affect the growth of bacterial cells but can inhibit or disperse biofilms, which are believed to reduce the emergency of “new drug resistance” (Majik and Parvatkar, 2014). The quorum sensing (QS) systems mediated by diffusible signaling molecules are among the most efficient regulatory mechanisms for *P. aeruginosa*, which regulate biofilm formation, secretion mechanisms, and the release of a large set of virulence factors such as pyocyanin (Lau et al., 2004a), elastase (Galloway, 1991), and rhamnolipids (Soberón-Chávez et al., 2005). A few classes of *P. aeruginosa* QS inhibitors (QSIs) have been shown to act as efficient biofilm inhibitors, including meta-bromo-thiolactone (mBTL) (O'loughlin et al., 2013), triazole-containing 2-phenylindole, salicylic acid (Srinivasarao et al., 2018), and 6-gingerol (Kim et al., 2015). However, most of the QSI compounds for *P. aeruginosa* were identified from synthetic compound libraries (Kalia, 2013), which may have the problem of high toxicity to the human body (Kalia et al., 2019). It makes sense to identify QSIs from microbial metabolites secreted by microorganisms colonizing the human body. *Pseudomonas aeruginosa* PQS can be found in the sputum of patients during infections (Pesci et al., 1999). After binding to its specific receptor PqsR, PQS can activate the expression of the *pqs* QS regulated genes (Soheili et al., 2019), including those responsible for the biosynthesis of pyocyanin and pyoverdine (García-Reyes et al., 2020). Therefore, blocking the *pqs* QS system will impair the ability of *P. aeruginosa* to form biofilms and produce virulence factors, thus attenuating infections.

In this study, Pht was shown to reduce the *pqsA-gfp* bioreporter expression in a dose-dependent manner in *P. aeruginosa*. Moreover, we showed that Pht can decrease biofilm formation and pyocyanin production of *P. aeruginosa*. The molecular docking analysis suggested that PQS and Pht bind to the same cavity of PqsR. The EMSA assay showed that Pht can inhibit PqsR protein from binding to the *pqsA* promoter. The production of the key *pqs* QS proteins, including PqsB, PqsD, and PqsA, was decreased by adding Pht into the bacterial culture according to the proteomic analysis. We further revealed that Pht analogs and vitamins K_1_, K_2_, and K_3_, could also inhibit the *P. aeruginosa pqs* QS system. Thus, our data suggested that the AhR ligand Pht and its vitamin K analogs are dual-functional molecules that can interfere with the *P. aeruginosa pqs* QS system and promote an AhR-dependent innate defense mechanism against bacteria.

## Materials and methods

### Cell cultivation

THP-1 (human monocytes, ATCC TIB-202) and THP-1 AhR reporter (Moura-Alves et al., 2019) cells were grown in RPMI 1,640 (GIBCO), supplemented with 10% (v/v) heat-inactivated fetal calf serum (FCS; GIBCO), and 1% (v/v) penicillin-streptomycin (GIBCO). All AhR reporter cell lines were maintained with an additional 5 mg/mL of Puromycin (Calbiochem). Cells were kept at 37°C in 5% CO_2_. Lentiviral infection was performed as described previously (Moura-Alves et al., 2019) and according to the protocols available on the RNAi Consortium website (https://portals.broadinstitute.org/gpp/public/). A similar protocol was used to generate the THP-1 Control and THP-1 AhR-Knockdown (KD) cell lines.

### RNA isolation and real-time quantitative-PCR

To quantify the expression of genes of interest, the THP-1 cells were grown in 24-well plates for 24 h with or without PQS/Pht/vitamin K addition. Total RNA was extracted using the TRIzol LS reagent (Invitrogen, California, USA). The qRT-PCR assay was performed using the ABI 7500 sequence detection system (Applied Biosystems, California, USA). Three replicates were performed with the glyceraldehyde-3-phosphate dehydrogenase gene (*GAPDH*) as the internal control gene.

### Molecular docking

The AhR and PqsR PDB structure files were downloaded from the Uniprot database and modified in PyMOL v. 1.4 (Schrodinger, LLC) (Seeliger and De Groot, 2010). The PQS, Pht, and vitamin K molecule files were downloaded from the Pubchem database. The docking process between proteins and potential ligands was performed in the Autodock Vina plug-in inside Chimera (Pettersen et al., 2004) (UCSF). The software program LIGPLOT v.4.5.3 (Wallace et al., 1995) was used to map the interactions between proteins and potential ligands. Open babel software (O'boyle et al., 2011) was used to transfer proper file formats.

### Microscale thermophoresis

The human recombinant AhR protein with Val128-Asn399 and N-terminal His-Tag was purchased from ImmunoClone (New York, USA). The binding of ligands to the AhR protein was assessed by microscale thermophoresis (MST) experiments using the Monolith^®^ NT.LabelFree (NanoTemper Technologies GmbH). MST measurements were performed according to the manufacturer's instructions previously described (Moura-Alves et al., 2019). The interaction affinity of the dissociation constant Kd for each ligand was analyzed using the NanoTemper Affinity software by the changes in the normalized fluorescence (fraction bound) vs. the ligand concentration.

### Bacterial strains and media

Bacterial strains and plasmids used in this study are described in Supplementary Table 1. Bacteria were cultured in Luria–Bertani (LB) broth (1% tryptone, 0.5% yeast extract, 0.5% NaCl) and ABTGC (B-medium (0.1% MgCl_2_, 0.1% CaCl_2_, 0.1% FeCl_3_) supplemented with 10% A10, 0.2% glucose, and 0.2% casamino acids). The media were solidified with 1.5% Bacto Agar (Difco). Kings' medium [Milli-Q water supplemented with proteose peptone (20 g/L), potassium sulfate (10 g/L), magnesium chloride, anhydrous (1.640 g/L), and glycerol (10% v/v)] were used for the pyocyanin quantification assay.

### *P. aeruginosa* mutant construction

*Pseudomonas aeruginosa* mutant strains Δ*pqsC* and Δ*pqsR* were generated by homologous recombination using the previously described protocol (Choi and Schweizer, 2005). The mutants were constructed by overlapping PCR to contain a gentamicin-resistance cassette. The mutant fragments were inserted into pK18, a suicide vector, to produce gene knockout plasmids. Each knockout plasmid was transformed into *E. coli* strain RK600 and conjugally transferred from RK600 to PAO1. The resultant integrants were selected on agar containing 30 μg/mL gentamicin (Gm30). To resolve merodiploids, we streaked the Gm-resistant colonies for single colonies on LB+Gm30 plates containing 5% sucrose. Potential mutants were screened by PCR using corresponding flanking primers and were confirmed by Sanger sequencing.

### *P. aeruginosa* QS inhibition and growth curve assays

PQS and Pht compounds were prepared in a 96-well microtiter plate (Nunc, Denmark) at a concentration of 100 mM in DMSO. PQS and Pht were then mixed with ABTGC medium and serial dilutions to give a concentration of 125, 250, 500, 750, 1,000, 1,500, and 2,000 nM, respectively. An overnight culture of the PAO1-*pqsA*-*gfp* strain (grown in LB medium at 37°C, 200 rpm) was diluted in ABTGC medium to a final optical density at 600 nm (OD_600nm_) of 0.02 (~2.5 × 10^8^ CFU/mL). A DMSO control (0.1% final concentration) and blank control (ABTGC medium) were used. The microtiter plate was incubated at 37°C in a Tecan Infinite 200 Pro plate reader (Tecan Group Ltd., Mannedorf, Switzerland) to measure the cell density (OD_600nm_) and GFP fluorescence (excitation at 485 nm, emission at 535 nm) at 30 min intervals for at least 12 h. The inhibition assay for PQS and Pht was done in triplicate. Growth curve assays were performed only by measuring the cell density (OD_600nm_) using the same method above.

### Pyocyanin quantification assay

Overnight cultures of *P. aeruginosa* WT and Δ*lasI*Δ*rhlI* mutant strains were standardized to an OD_600nm_ of 0.1 and diluted 100 times into 25 mL of fresh King's Medium in a 250 mL flask. The PQS and Pht compounds were added to give a final concentration of 750 nM. The cultures were grown for 48 h at 37°C in shaking conditions (200 rpm). The cultures were monitored every 24 h to observe any color change from light-yellow to greenish-yellow in the untreated flask. The mutant strain Δ*lasI*Δ*rhlI* was used as the negative control. The final cell density was measured at 600 nm (OD_600nm_) using a Tecan Infinate 200 Pro plate reader (Tecan Group Ltd., Mannedorf, Switzerland). The cultures were then centrifuged for 10 min at 10,000 rpm, and 7.5 mL of the supernatants were transferred into new Falcon tubes. Pyocyanin extraction was conducted using chloroform (3 mL) and 0.2 M HCl (1.5 mL). The top aqueous layer of HCl containing pyocyanin was pipetted into a microtiter plate, and absorbance was measured at 520 nm. The data was normalized by dividing the OD_520nm_ reading by the final OD_600nm_ values.

### Biofilm formation assay

Biofilm formation was quantified by crystal violet staining. Overnight LB-grown cultures were diluted in fresh medium (1:100) and incubated in 2 mL of LB in 15 mL tubes at 37°C for 24 h, statically allowing biofilm formation. After removing the spent media, we removed the loosely associated bacteria by washing them with water two times, and the remaining bacteria were stained with 0.1% crystal violet for 20 min. Then, the crystal violet stain was discarded while the stained biofilms were washed twice thoroughly with ddH_2_O and air-dried. The stained biofilm was then dissolved into 200 μL of 95% ethanol and quantified at 630 nm using a Tecan Infinate 200 Pro plate reader (Tecan Group Ltd., Mannedorf, Switzerland).

### The electrophoretic mobility shift assay

EMSA assays were performed using cell lysates of PAO1 and *pqsR* mutants as previously described (Gruber et al., 2016). For each sample, cell-lysate was incubated with biotinylated 248-bp *pqsA* promoter DNA (pqsA′), which was generated by PCR using biotinylated forward primer (TTCTTGCTTGGTTGCCG) and reverse primer (GACAGAACGTTCCCTCTT). The reaction mixture contained 10 mM Tris–HCl (pH 8.0), 1 mM EDTA, 50 mM NaCl, 1 mM DTT, 1 μg/μL Poly (dI-dC) and was incubated at room temperature (24°C) for 20 min. Samples were separated on a 5% polyacrylamide gel in 0.5 × Tris Borate EDTA (TBE) run at 100 V for 120 min and transferred onto a positively-charged nylon membrane at 40 V for 1 h. Biotinylated DNA on the nylon membrane was probed by streptavidin-HRP conjugate, detected by a chemiluminescent substrate (Beyotime EMSA kit; Shanghai, China), and visualized by exposing it to a GE ImageQuant RT-ECL instrument.

### Tandem mass tag-based quantitative proteomic analysis

Overnight PAO1 cultures were diluted 100 times into 25 mL of fresh LB medium. A Pht compound was added to one sample to give a final concentration of 750 nM. Then, total proteins were extracted from bacterial samples and trypsin digested. Then, the digested sample was labeled with a TMT reagent. Next, TMT labeled peptide was separated by a RIGOL L-3000 dual gradient High-Performance Liquid Chromatography (HPLC) using an Agela Durashell-C18 column. An HPLC-separated peptide was identified by nano UPLC-MS/MS consisting of a Nanoflow HPLC system (EASY-nLC 1000 system from Thermo Scientific) with an EASY-Spray C18 column and an Orbitrap Fusion Lumos mass spectrometer (Thermo Scientific). Then, the protein was identified using the Uniprot_HUMAN database. The resulting MS/MS data were processed using Proteome Discoverer 2.4. The final results were functionally annotated by Gene Ontology (GO) (Zhang et al., 2019), KEGG (https://www.genome.jp/kegg/) (Jones et al., 2014), and STRING (https://www.string-db.org) (Franceschini et al., 2013).

### Statistical analyses

All statistical analyses were performed using GraphPad Prism software with a two-sided Mann-Whitney *U*-test and Benjamini-Hochberg-corrected *P-*values. Data represent the mean ± standard deviation (SD) of three independent experiments unless otherwise indicated. *P-*values < 0.05 were considered statistically significant. All false discovery rate controls were performed with the Benjamini-Hochberg procedure, and a false-discovery rate of 10% (*q* < 0.1) was selected as the significant threshold. Statistical details for all tests performed can be found in the figure legends.

## Results

### PQS inhibited while Pht activated AhR downstream genes *CYP1B1* and *AHRR* in THP-1 human monocytes

**Figure 1:**
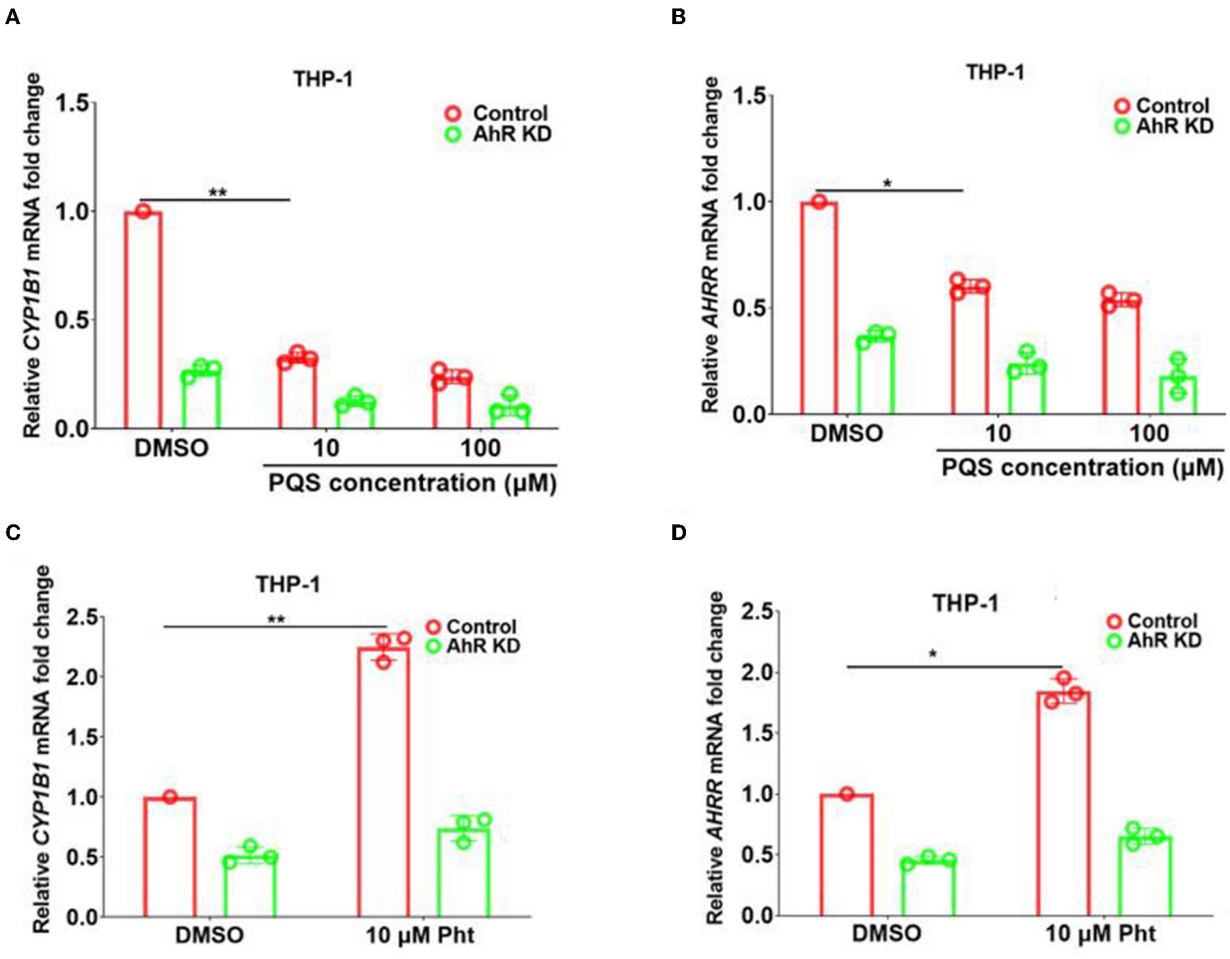
PQS inhibited while Pht activated AhR downstream genes in THP-1 human monocytes. **(A,B)** qRT-PCR quantification of the expression of *CYP1B1* and *AHRR* in THP-1 Control and AhR KD cells with PQS addition. **(C,D)** qRT-PCR quantification of the expression of *CYP1B1* and *AHRR* in THP-1 Control and AhR KD cells with Pht addition. Data are presented as means ± SD; *n* = three independent experiments (*P ≤ 0.05 and **P ≤ 0.01, Mann-Whitney *U*-test).

*Pseudomonas aeruginosa* QS signal molecule PQS has been reported to downregulate AhR-induced cytochrome P4501 (CYP1) enzyme and CYP1A1 enzymatic activities in mouse liver cells (Hepa-1c1c7) (Moura-Alves et al., 2019). We demonstrated by qRT-PCR that the transcription of two AhR downstream genes, *CYP1B1* and AhR repressor (*AHRR*), was repressed 2.3- and 2.5-fold with the addition of 10 μM PQS in THP-1 human monocytes compared to control (additional of DMSO alone) (Figures 1A,B). In addition, there were no significant expression changes of these genes in the THP-1 KD cells upon PQS addition.

The MTb natural metabolite Pht was found to activate *CYP1B1* and *AHRR* transcription in THP-1 cells in an AhR-dependent manner (Moura-Alves et al., 2014). Our qRT-PCR results also confirmed that *CYP1B1* and *AHRR* transcription were promoted by 2.2- and 1.9-fold with the addition of 10 μM Pht in THP-1 human monocytes compared to control (DMSO only), respectively (Figures 1C,D). These results demonstrated that PQS and Pht adversely affected AhR regulatory pathways.

### *In vitro* assays of PQS or Pht interactions with AhR

**Figure 2:**
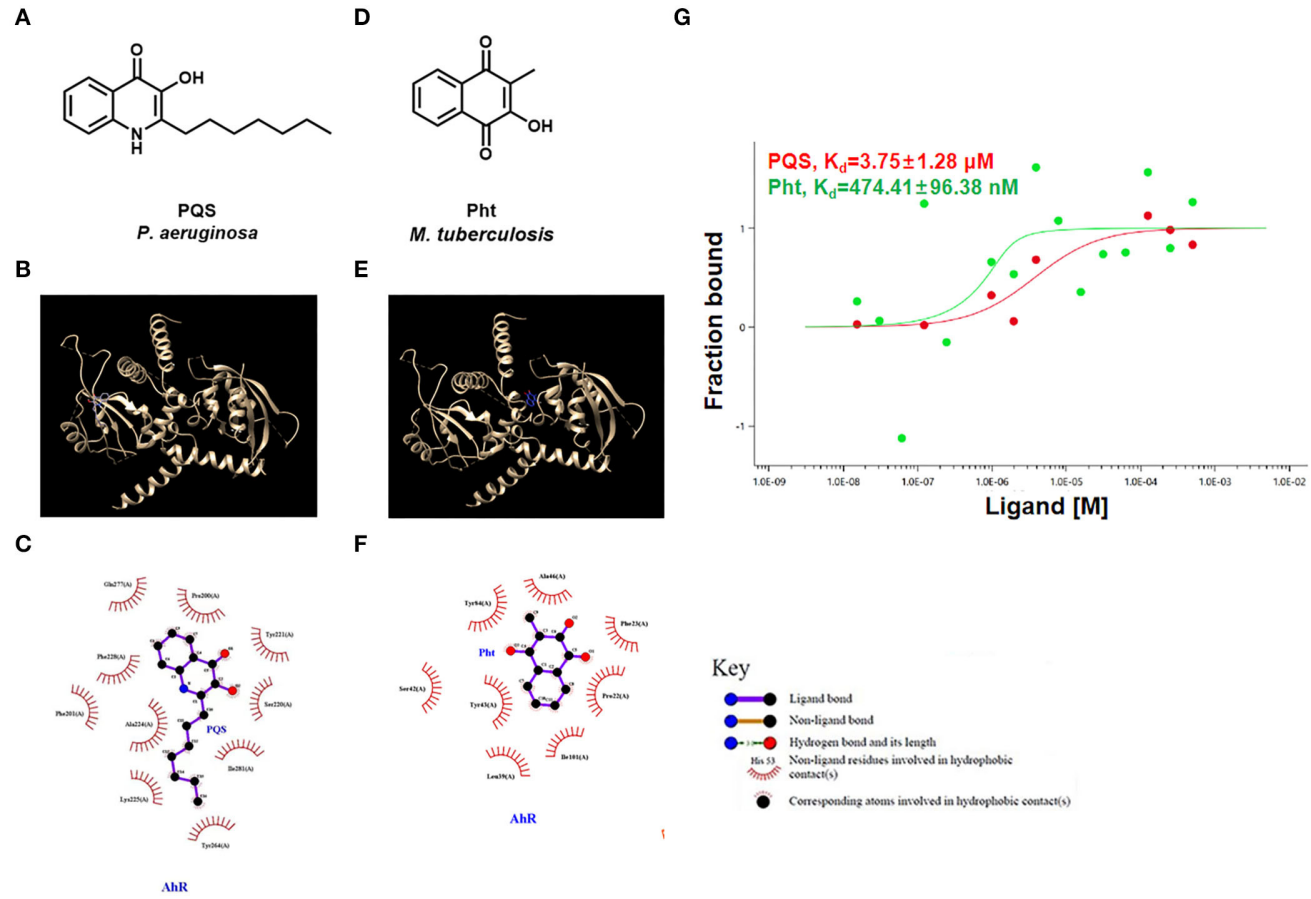
PQS and Pht were able to bind to AhR protein. **(A,D)** Molecular structures of *P. aeruginosa* PQS and MTb Pht. **(B,E)** The interaction model of the AhR protein with PQS or Pht. **(C,F)** Interaction map between residues within the AhR protein with PQS or Pht. **(G)** Quantifying the binding affinity of AhR protein with PQS or Pht using microscale thermophoresis assay.

The chemical structures of the *P. aeruginosa* auto-inducer PQS and MTb Pht are shown in Figures 2A,D. Molecular docking was performed using the ligands PQS and Pht against the ligand-binding domain of the human AhR protein. These compounds and their structures are shown in Figures 2B,E. To map the interactions between PQS and the residues within the ligand-binding site on AhR, we used the software LIGPLOT v. 4.5.3 (Wallace et al., 1995). This program provides a 2-dimensional map showing the hydrogen-bond and hydrophobic interactions between atoms in the ligand and those of the binding partner.

The results showed that PQS binds to the AhR protein through hydrophobic forces, and they appear to interact at residues Gln 277, Tyr 221, Pro 200, Ile 281, Ser 220, Phe 228, Phe 201, Ala 224, Lys 225, and Tyr 264 (Figure 2C). However, the docking analysis suggested that Pht and AhR appeared to interact at residues Tyr 84, Ala 46, Ile 101, Pro 22, Phe 23, Tyr 43, Ser 42, and Leu 39 (Figure 2F).

Next, we used an MST assay to quantify the AhR binding affinity with different ligands, including PQS and Pht. The MST results indicated that the dissociation constant (Kd) values of AhR protein with PQS and Pht were 3.75 μM and 474.41 nM, respectively (Figure 2G). These results confirmed that PQS and Pht directly bind to AhR protein as ligands.

### Effects of Pht on *P. aeruginosa pqs* QS system, pyocyanin production, and biofilm formation

To engineer a PQS auto-inducer reporter system, we first made a PQS deficient Δ*pqsC* mutant by deleting the *pqsC* gene. The *pqsA*-*gfp* reporter plasmid (Yang et al., 2007) was next transferred into the Δ*pqsC* mutant to make the PQS biosensor strain Δ*pqsC* P_pqsA−*gfp*_. In addition, the *pqsA*-*gfp* reporter plasmid was transferred into the PAO1 WT to make the PQS bioreporter strain PAO1 P_pqsA−*gfp*_. Induction of the *pqsA* gene is mainly controlled by PqsR (the signal receptor of the *pqs* QS system), which is activated by the presence of PQS (Soheili et al., 2019). Thus, in the PAO1 P_pqsA−*gfp*_ strain, a decrease in GFP fluorescence would indicate the presence of an antagonist of PQS, while exogenous PQS was needed to induce the GFP fluorescence of the Δ*pqsC* P_pqsA−*gfp*_ strain.

**Figure 3:**
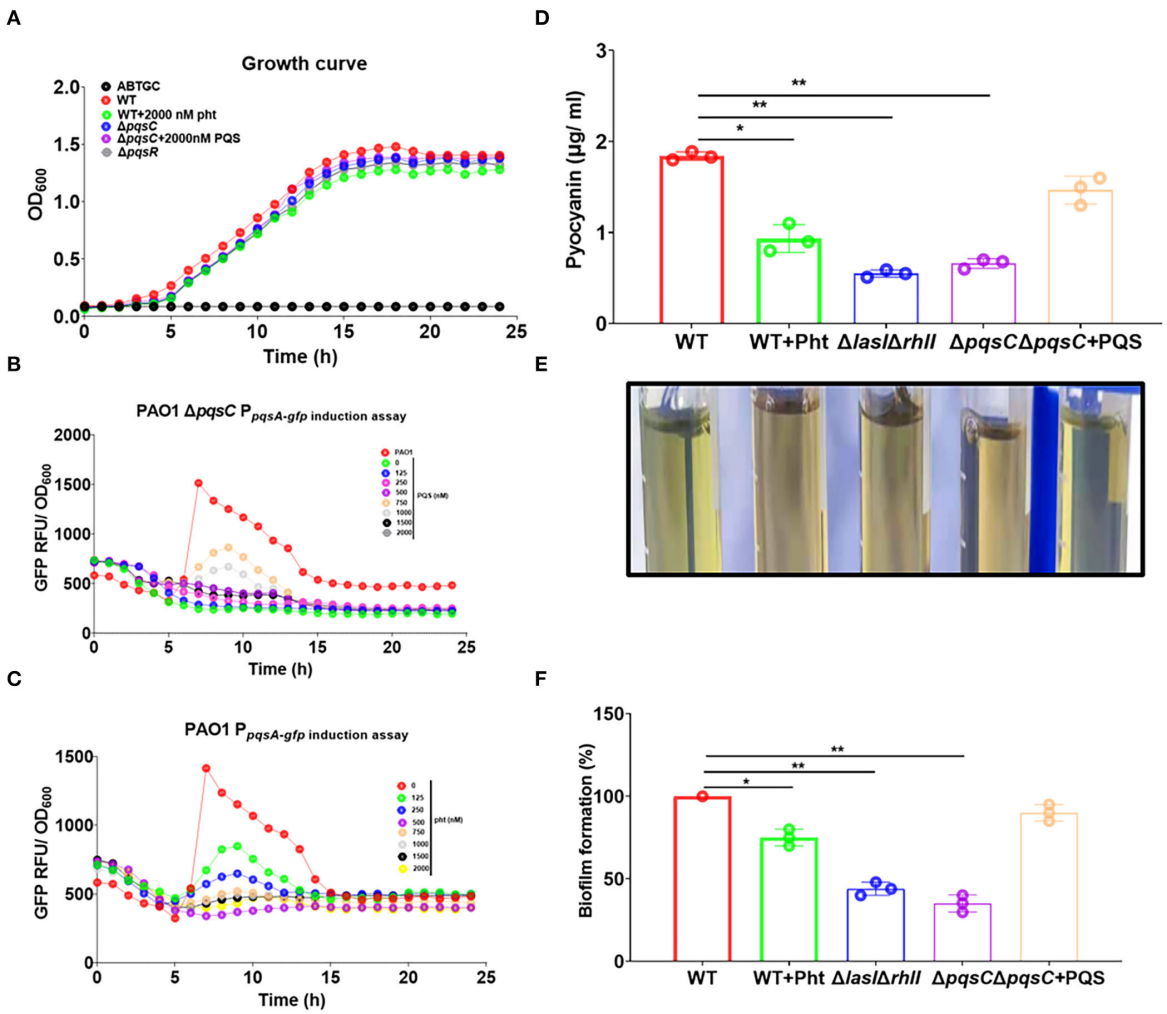
Pht inhibited *P. aeruginosa pqs* QS system, pyocyanin production, and biofilm formation. **(A)** Growth curves of *P. aeruginosa* PAO1 WT and QS mutant strain under different conditions. **(B)** Dose-dependent induction curves of PQS in Δ*pqsC* P_pqsA−*gfp*_. **(C)** Dose-dependent inhibition curves of Pht in PAO1 P_pqsA−*gfp*_. **(D)** The production of pyocyanin by *P. aeruginosa* WT, WT+750 nM Pht, Δ*lasI*Δ*rhlI*, Δ*pqsC*, Δ*pqsC*+750 nM PQS strains. **(E)** Photos of visualization of the green pigment corresponding to the upper panel. **(F)** The biofilm formation of *P. aeruginosa* WT, WT+750 nM Pht, Δ*lasI*Δ*rhlI*, Δ*pqsC*, Δ*pqsC*+750 nM PQS strains. Data are presented as means ± SD; *n* = three independent experiments (*P ≤ 0.05 and **P ≤ 0.01, Mann-Whitney *U*-test).

Pht was exogenously added to both the above reporter strains to evaluate its impact on the *pqs* QS system. As shown in Figure 3A, both PQS and Pht could not affect the growth of the *P. aeruginosa* strain. PQS—not Pht—induced the *pqsA*-*gfp* expression in a dose-dependent manner in the PAO1 Δ*pqsC* strain (Figure 3B). In contrast, Pht was able to inhibit *pqsA*-*gfp* expression in a dose-dependent manner in the PAO1 P_pqsA−*gfp*_ strain, with the optimal effect at a concentration of 750 nM (Figure 3C). These results showed that PQS activated while Pht inhibited the *P. aeruginosa pqs* QS system.

The production of pyocyanin and the formation of biofilms are *pqs* QS system-regulated phenotypes related to virulence in *P. aeruginosa* (Pearson et al., 1997). Pyocyanin is a key virulence factor produced by *P. aeruginosa* that plays a major role in cystic fibrosis (CF) lung infections (Lau et al., 2004a). Pyocyanin was shown to cause tissue damage and necrosis in a *P. aeruginosa* murine model for a lung infection (Lau et al., 2004b). Biofilm formation by *P. aeruginosa* is well known as the cause of chronic infections (Costerton et al., 2005). We thus evaluated the effects of Pht on these two phenotypes.

As expected, the pyocyanin production by *P. aeruginosa* PAO1 strain was found to be suppressed by Pht to a level comparable to that of PAO1 Δ*lasI*Δ*rhlI* strain and the Δ*pqsC* strain. It could be seen from the intensity of the green pigment in the supernatants of different samples that the PAO1 WT culture exhibited a dark green, Δ*lasI*Δ*rhlI* and Δ*pqsC* cultures exhibited light green, and the WT+Pht culture did not have a visible green color. These results indicated that Pht inhibited pyocyanin production (Figure 3D). Crystal violet staining showed that the biofilm formation of *P. aeruginosa* PAO1 WT was suppressed by Pht to a level comparable to that of the PAO1 Δ*lasI*Δ*rhlI* and the Δ*pqsC* strains (Figures 3E,F). Besides, we detected that Pht did not inhibit *P. aeruginosa* rhamnolipid productions (Supplementary Figure 1). These results indicated that Pht repressed *P. aeruginosa pqs* QS system, pyocyanin production, and biofilm formation.

### Investigation of the interaction of Pht with PqsR protein

**Figure 4:**
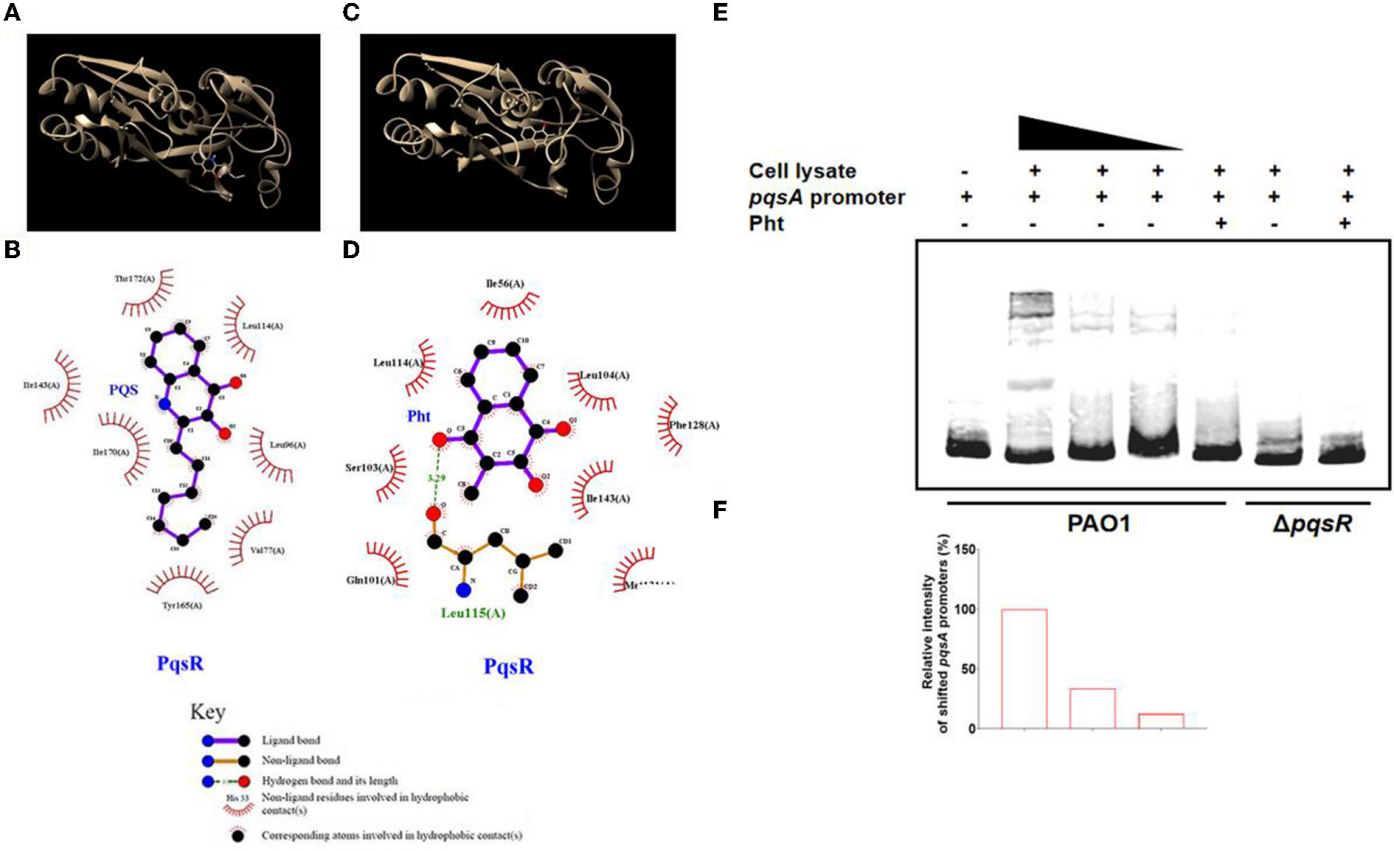
Interaction of Pht with PqsR protein. Molecular docking of the PqsR protein with PQS or Pht. **(A,C)** The interaction model of the PqsR protein with PQS or Pht. **(B,D)** Interaction map between residues within the PqsR protein with PQS or Pht. **(E)** EMSA assays with biotinylated-*pqsA* promoters incubated with cell-lysates of PAO1 and Δ*pqsR* strains. **(F)** The abundance of shifted cell-lysates corresponding to the upper panel was determined by measuring the densitometry in Image J.

The interactions between alkyl quinolones with PqsR are simulated by the crystal structure of PqsR complexed with its ligand, which suggests the influence of ligand binding on PqsR affinity to its DNA-binding site (Ilangovan et al., 2013). Molecular docking was first performed using the reference ligand PQS and potential ligand Pht against the ligand-binding domain of the *P. aeruginosa* PqsR protein. These compounds and their structures were shown (Figures 4A,C). To map the interactions between PQS and the residues within the ligand-binding site on PqsR, the software program LIGPLOT v. 4.5.3 was used.

The results showed that PQS binds to PqsR protein by hydrophobic forces, and they appeared to interact at residues Leu 189, Tyr 258, Val 170, Ile 236, Ile 149, Ala 168, Thr 265, and Leu 207 (Figure 4B). While the docking analysis suggested that Pht and PqsR appeared to interact at residues Ile 56, Leu 114, r103 (A), n101 (A), Met 131, Ile 143, Phe 128, and Leu 104 (Figure 4D). Except for hydrophobic forces, Pht and PqsR are also predicted to be bound by a hydrogen bond at Leu 115 (A). These molecular docking results revealed that the QS signaling molecule PQS and Pht competed for the same active pocket in the PqsR protein. The hydrogen bond between Pht and PqsR might offer Pht an advantage when competing with PQS. Thus, we suspected that Pht competitively disrupted the PQS-mediated PqsR activation and signaling in *P. aeruginosa*. These predicted binding modes of PqsR and Pht need further experimental verification.

The electrophoretic mobility shift assay (EMSA) is a rapid and sensitive experimental method to detect protein-nucleic acid interactions (Hellman and Fried, 2007). The protein-nucleic acid complexes migrate more slowly than the corresponding free nucleic acid in the gel. To test whether Pht will interact with PqsR and further affect its DNA binding capacity, we performed EMSA with the biotinylated-*pqsA* promoter (Bio-*pqsA*) using cell lysates of PAO1 WT and Δ*pqsR* strains. The results showed that only PAO1 cell lysates shifted Bio-*pqsA* migration, while Δ*pqsR* cell lysates did not show any effect. The addition of Pht attenuated the interaction between PAO1 cell lysates and Bio-*pqsA*, whereas there was no effect by adding the cell lysates of Δ*pqsR* (Figures 4E,F). These results indicated that Pht could indeed block the binding of PqsR protein to the *pqsA* promoter, which further leads to the inhibitory effect against *P. aeruginosa pqs* QS systems.

### Pht down-regulated the expression of *pqs* QS regulated proteome

To study the changes in the proteomic profile of *P. aeruginosa* PAO1 upon Pht addition, TMT was used as the labeling strategy for comparative quantitative proteomic analysis (performed with a false discovery rate below 1%). The following cutoffs were used for protein identification: an unused protein score of at least 2 (i.e., 99% confidence of identification) and having more than 1 peptide identified. Using these cutoffs, we identified 2,203 proteins. Using a *p*-value cutoff of 0.05, 37 proteins were found to be significantly affected by Pht. The expression of 10 proteins was upregulated, whereas the expression of 27 proteins was downregulated (Supplementary Table 2).

Table 1 shows the 27 proteins whose abundances were significantly decreased in the Pht-treated *P. aeruginosa* PAO1 strain compared to those in the control PAO1 strain without Pht addition. Of these 27 proteins, five have been previously found to be *pqs* QS regulated: *phnA* (Anthranilate synthase component 1), *pqsB* (Anthraniloyl-CoA anthraniloyltransferase), *phzD2* (Phenazine biosynthesis protein PhzD2), *pqsD* (Anthraniloyl-CoA anthraniloyltransferase), and *pqsA* (Anthranilate–CoA ligase). These results further confirmed that Pht directly attenuates *P. aeruginosa pqs* QS regulated global proteome network.

**Table 1:** Protein expression changed in PAO1 WT with the addition of Pht^a^.

**Gene name**	**Protein description**	**Protein expression fold change**
**Down-regulated:**
*pqsA*	Anthranilate–CoA ligase	0.84
PA2379	Probable oxidoreductase	0.83
PA4046	Uncharacterized protein	0.83
*tatA*	Sec-independent protein translocase protein TatA	0.83
*uvrC*	UvrABC system protein C	0.83
PA5220	Uncharacterized protein	0.83
*pqsD*	Anthraniloyl-CoA anthraniloyltransferase	0.83
PA1668	DotU domain-containing protein	0.82
PA0752	Uncharacterized protein	0.82
*sdhC*	Succinate dehydrogenase cytochrome b556 subunit	0.81
PA3331	Cytochrome P450	0.81
*nusG*	Transcription termination/antitermination protein NusG	0.81
PA4357	FeoC domain-containing protein	0.81
*hcnC*	Hydrogen cyanide synthase subunit HcnC	0.81
*pilP*	Type IV pilus inner membrane component PilP	0.81
*citA*	Citrate transporter	0.80
*pmrB*	Sensor protein kinase PmrB	0.80
PA4591	HlyD_D23 domain-containing protein	0.80
PA2526	Probable Resistance-Nodulation-Cell Division (RND) efflux transporter	0.79
PA1655	Probable glutathione S-transferase	0.79
PA2252	Probable AGCS sodium/alanine/glycine symporter	0.78
*pilO*	Type IV pilus inner membrane component PilO	0.77
*phnA*	Anthranilate synthase component 1, pyocyanin specific	0.76
PA1654	Probable aminotransferase	0.76
*wspC*	Probable biofilm formation methyltransferase WspC	0.76
*roxS*	Histidine kinase	0.75
PA3677	Probable Resistance-Nodulation-Cell Division (RND) efflux membrane fusion protein	0.72
*phzD2*	Phenazine biosynthesis protein PhzD2	0.68
*purE*	N5-carboxyaminoimidazole ribonucleotide mutase	0.64
*pqsB*	2-heptyl-4(1H)-quinolone synthase subunit PqsB	0.59
**Up-regulated:**
PA1224	Probable NAD(P)H dehydrogenase	1.56
*fabZ*	3-hydroxyacyl-[acyl-carrier-protein] dehydratase FabZ	1.39
*ribH*	6,7-dimethyl-8-ribityllumazine synthase	1.33
PA5076	Probable binding protein component of ABC transporter	1.32
PA4880	Probable bacterioferritin	1.29
PA4739	BON domain-containing protein	1.25
*nalC*	NalC	1.23
*speD*	S-adenosylmethionine decarboxylase proenzyme	1.22
*ihfB*	Integration host factor subunit beta	1.22
PA3779	Uncharacterized protein	1.22

^a^Three replicates per sample in the log phase were performed for proteomics analysis.

### Vitamins K_1_ upregulated AhR downstream genes *CYP1B1* and *AHRR* through AhR in THP-1 human monocytes

**Figure 5:**
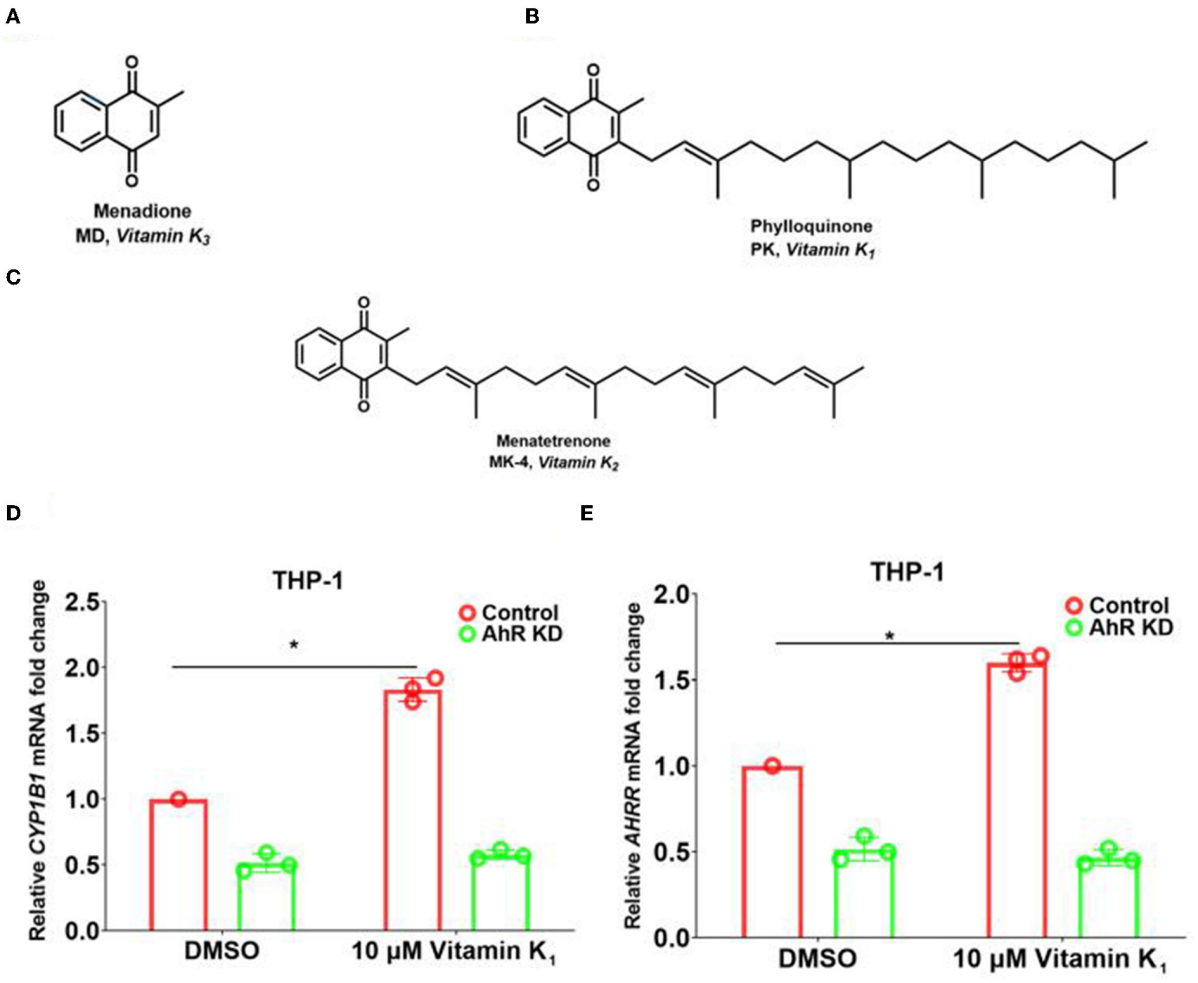
Pht analog vitamins K_1_ increased the expression of AhR downstream genes in THP-1 human monocytes through AhR. **(A–C)** The chemical construction of molecules of vitamins K_1_, K_2_, and K_3_. **(D,E)** qRT-PCR of the expression of *CYP1B1* and *AHRR* in THP-1 control and AhR KD cells with vitamin K_1_ addition. Data are presented as means ± SD; *n* = three independent experiments (*P ≤ 0.05, Mann-Whitney *U*-test).

By comparing the online PubChem compound library (https://pubchem.ncbi.nlm.nih.gov/), we found several structural analogs of the Pht molecule, including vitamins K_1_, K_2_, and K_3_ (Figures 5A–C). Vitamin K is a fat-soluble vitamin that participates in blood clotting (Palermo et al., 2017). Vitamin K deficiency may lead to prolonged blood clotting time, bleeding, and even death in severe cases (Palermo et al., 2017). Vitamin K not only has protective effects on the heart and the cerebrovascular system but also has a maintenance effect on the normal elasticity of blood vessel walls and participates in the metabolism of bone for the prevention and cure of osteoporosis and fracture (Fusaro et al., 2020). However, no evidence has yet shown that vitamin K can directly affect bacterial pathogenesis.

To reveal whether Pht analogs vitamin K families influence the AhR pathway, we performed qRT-PCR experiments to measure the transcription of AhR downstream genes. The results showed that *CYP1B1* and *AHRR* transcription was increased by 1.9- and 1.6-fold with the addition of 10 μM vitamin K_1_ in THP-1 human monocytes compared to that with DMSO (Figures 5D,E). Besides, there is no change in AhR KD cells with vitamin K_1_ stimulation. These results indicated that vitamin K_1_ promoted AhR downstream genes *CYP1B1* and *AHRR* through AhR in THP-1 human monocytes.

### *In vitro* assays examining the interactions of vitamin K with AhR protein

**Figure 6:**
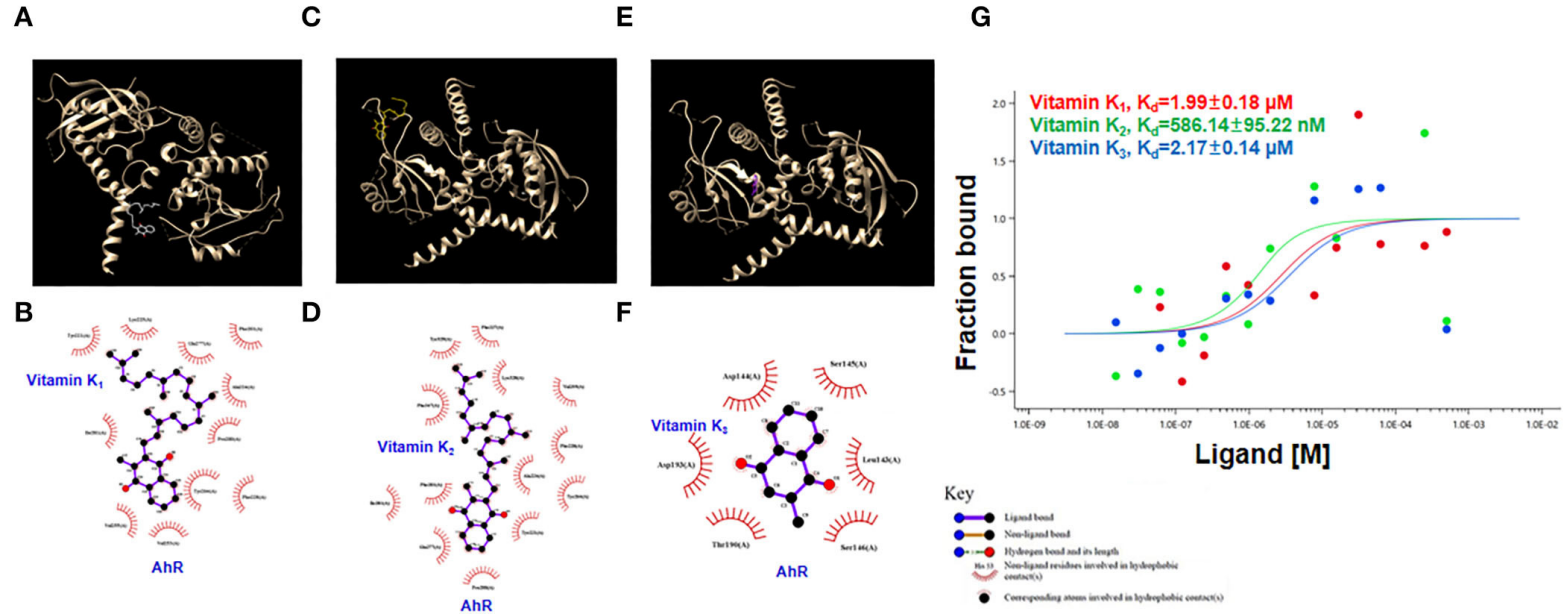
Interaction of vitamins K_1_, K_2_, and K_3_ with AhR protein. **(A,C,E)** The interaction model of the AhR protein with vitamins K_1_, K_2_, or K_3_. **(B,D,F)** Interaction map between residues within the AhR protein with vitamins K_1_, K_2_, or K_3_. **(G)** Quantification of the binding affinity of AhR protein with vitamins K_1_, K_2_, or K_3_ using microscale thermophoresis assay.

Molecular docking was performed using the ligands vitamins K_1_, K_2_, and K_3_ against the ligand-binding domain of the human AhR protein. Figures 6A,C,E show these compounds and their structures. The LIGPLOT results showed that vitamin K_1_ and AhR protein were bound by hydrophobic forces that appeared to interact at residues Tyr 221, Lys 225, Gln 277, Phe 201, Ala 224, Pro 200, Tyr 264, Phe 228, Val 253, Val 255, and Ile 281 (Figure 6B). Similarly, the docking analysis suggested that vitamins K_2_ and K_3_ with AhR appeared to interact at several vital residues (Figures 6D,F).

We further used the MST assay to detect AhR protein binding with vitamins K_1_, K_2_, and K_3_. The MST results indicated that the dissociation constant (Kd) values of AhR protein with vitamins K_1_, K_2_, and K_3_ were 1.99 μM, 586.14 nM, and 2.17 μM, respectively (Figure 6G). These findings showed that vitamins K_1_, K_2_, and K_3_ were able to bind AhR and are likely to modulate its activity.

### Effects of Pht analog vitamins K_1_, K_2_, and K_3_ on the *P. aeruginosa pqs* QS system

**Figure 7:**
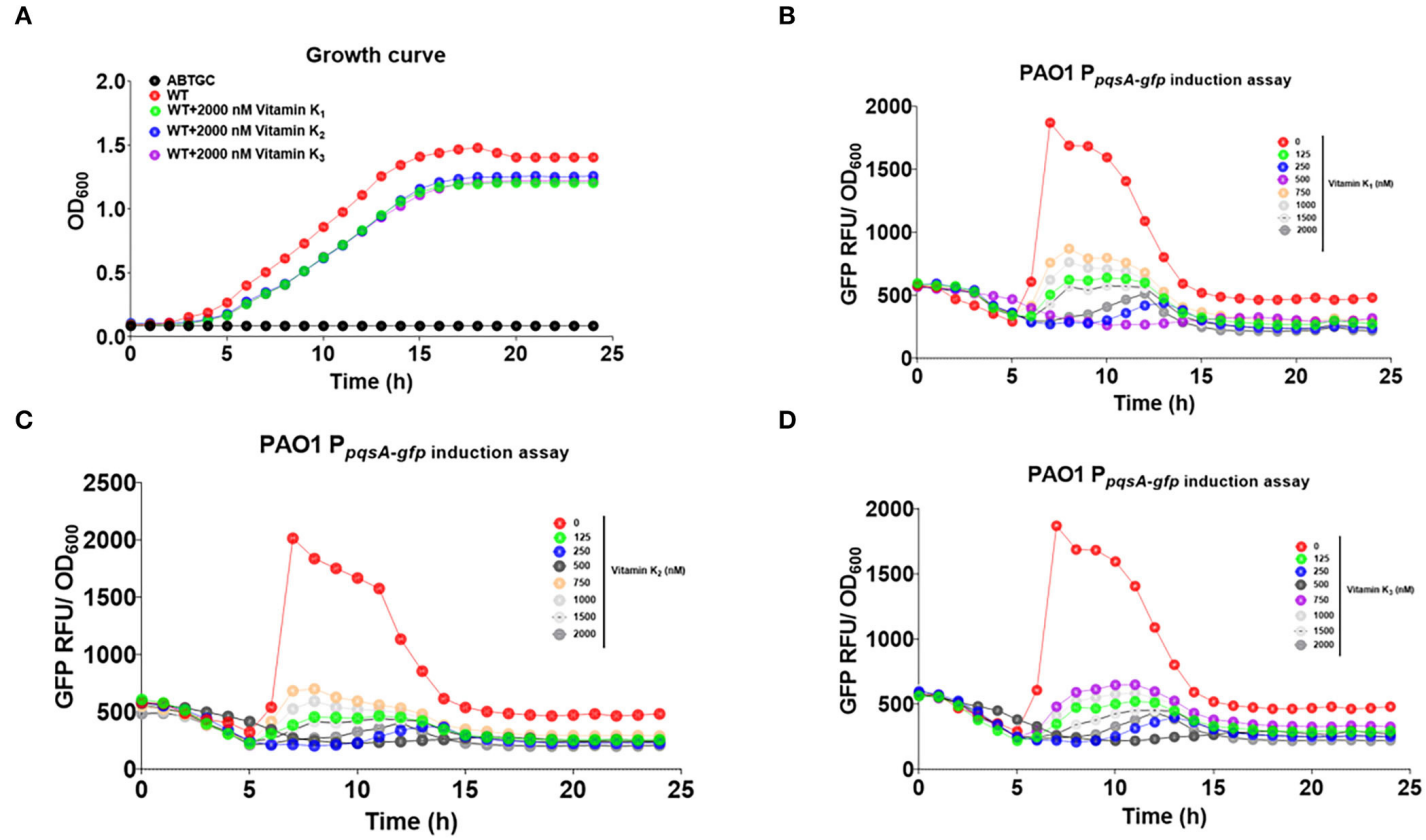
Vitamins K_1_, K_2_, and K_3_ inhibited *P. aeruginosa pqs* QS system. **(A)** Growth curve of ABTGC media (Blank), *P. aeruginosa* WT, WT+2000 nM vitamins K_1_, K_2_, and K_3_ strains. **(B)** Dose-dependent inhibition curves of vitamins K_1_ with QS monitor PAO1 P_pqsA−*gfp*_. **(C)** Dose-dependent inhibition curves of vitamins K_2_ with QS monitor PAO1 P_pqsA−*gfp*_. **(D)** Dose-dependent inhibition curves of vitamins K_3_ with QS monitor PAO1 P_pqsA−*gfp*_.

We then tested whether Pht analogs, vitamins K1, K2, and K3, had an inhibitory effect on the *P. aeruginosa pqs* QS system. The growth of the reporter strain (OD_600nm_) was monitored to ensure that vitamins K_1_, K_2_, or K_3_ would not affect the growth of *P. aeruginosa* strains (Figure 7A). GFP expression was normalized by dividing the GFP values by the OD values measured at respective time points. Vitamin K_1_, K_2_, and K_3_ were found to inhibit *pqsA*-*gfp* expression dose-dependently without affecting growth (Figures 7B–D). In conclusion, Pht and its analogs, vitamins K1, K2, and K3, inhibited the *P. aeruginosa pqs* QS system. In addition, we performed molecular docking to predict the binding modes of vitamins K_1_, K_2_, and K_3_ to PqsR protein (Supplementary Figure 2).

## Discussion

QSI compounds are effective agents for inhibiting *P. aeruginosa* virulence and biofilms, and the mode of action of some QSIs has been extensively investigated *in vivo* (Kalia et al., 2019). For example, the sulfur-containing compound ajoene (4,5,9-trithiadodeca-1,6,11-triene 9-oxide) purified from garlic extract was shown *in vitro* to significantly inhibit a subclass of QS-regulated *P. aeruginosa* genes and rhamnolipid production in a dose-dependent manner. Besides, in a murine pulmonary infection model, a significant clearing of infecting *P. aeruginosa* was detected in ajoene-treated mice compared to a nontreated control group (Jakobsen et al., 2012). Additionally, mBTL is a chemical molecule synthesized to inhibit the two *P. aeruginosa* QS regulators, LasR and RhlR, which were shown to reduce the production of the virulence factor pyocyanin and biofilm formation, protecting *Caenorhabditis elegans* from killing by *P. aeruginosa* (O'loughlin et al., 2013). However, most QSIs are discovered from screening chemical compound libraries and may be highly toxic to the human body. Therefore, it is urgent to discover and develop harmless QSI compounds such as microbial metabolites that could be detected in human lung tissues at high concentrations.

**Figure 8:**
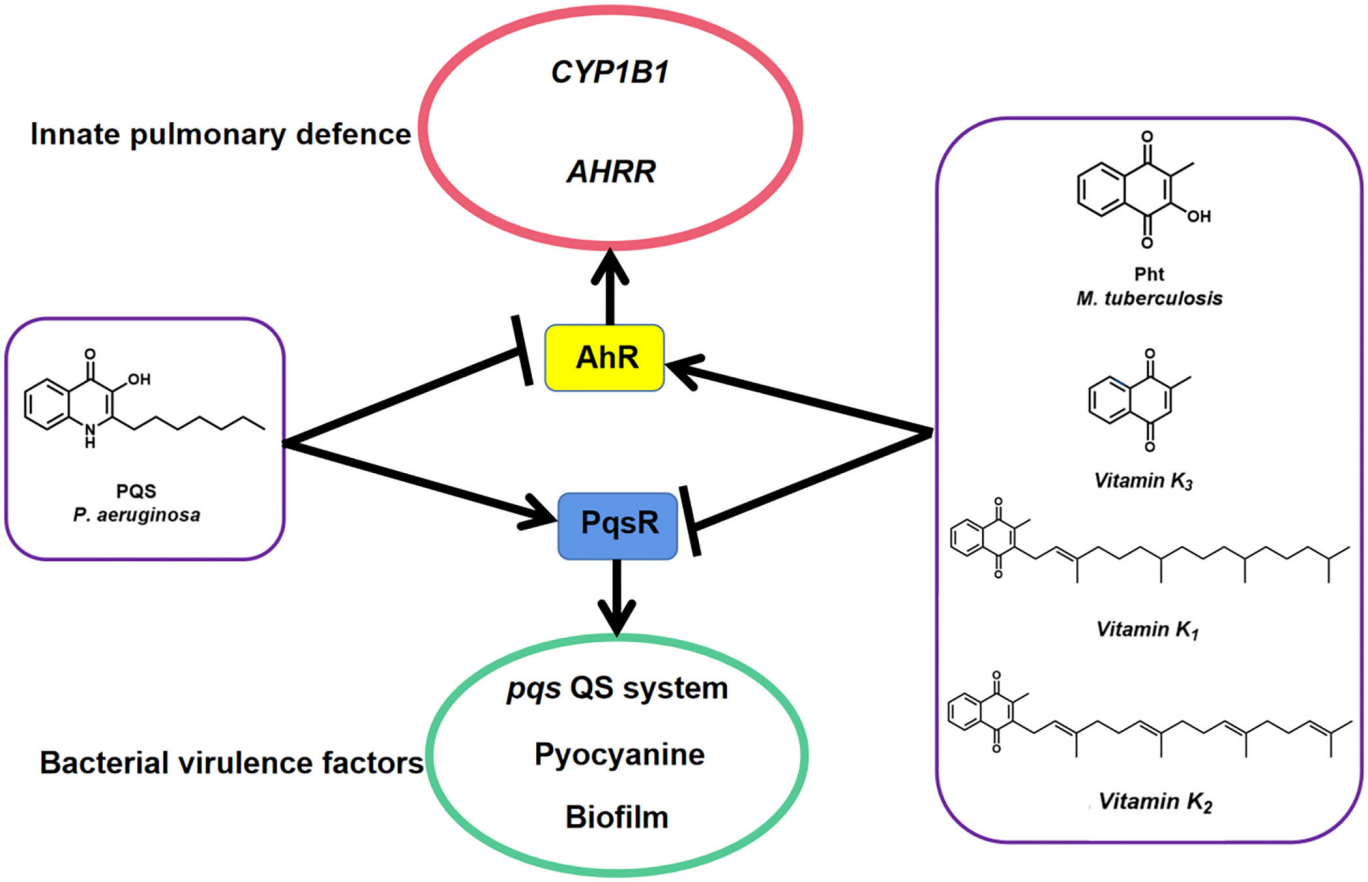
A model illustrates how *P. aeruginosa* PqsR and host protein AhR respond to MTb natural metabolites PQS and Pht. Pht and its analog vitamins K promote AhR to facilitate innate pulmonary defense against bacteria through *CYP1B1* and *AHRR* while repressed PqsR to block bacterial virulence factors such as *pqs* QS system, pyocyanin, and biofilm. Data suggest that PQS has the opposite effect.

Recently, one MTb natural metabolite Pht analog Plumbagin secreted by *Plumbago zeylanica* L. was identified to inhibit *P. aeruginosa* LasR protein-regulated *las* QS system (Qais et al., 2021). In this study, we reported the dual-functional *P. aeruginosa* PQS and MTb Pht interact with host AhR and bacterial PqsR proteins (Figure 8). Pht and its analog, vitamin K, promote AhR to facilitate innate immune defense against bacteria through *CYP1B1* and *AHRR*, while PQS had the opposite effect. Moreover, Pht and its analogs, vitamins K_1_, K_2_, and K_3_, were also effective QSIs that can inhibit *P. aeruginosa pqs* QS system, pyocyanin, and biofilm. Pht has been detected in the bronchoalveolar fluid with a concentration of up to 50 μM in most patients with tuberculosis (Gardner, 1996). The toxicity of Pht was previously tested in mice at a dose of 200 mg/kg oral and 150 mg/kg intraperitoneal, which is believed to be safe for *in vivo* usage (https://pubchem.ncbi.nlm.nih.gov/compound/10221). Besides, Pht analogs and vitamin K also naturally exist in the human body and have been proven to boost the human immune system by preventing calcium deposition, elastic fiber degradation, thrombosis, and inflammation in blood vessels or lungs (Janssen et al., 2021). The vitamin K family has been approved by the FDA and long-term used as human drugs and healthy foods without toxicity. Thus, Pht and Vitamin K could be potentially harmless QSI drugs for controlling *P. aeruginosa* infections and increasing human immune systems.

Carbapenems, including imipenem, meropenem, and ertapenem, are common antibiotics to treat *P. aeruginosa* biofilm-associated infections in patients with CF (Yan and Bassler, 2019). However, due to the long-term use of these antibiotics, *P. aeruginosa* evolved multidrug resistance mechanisms to them, such as expression of efflux pumps and overexpression of biofilm structural components (Hall and Mah, 2017). Specifically, QSIs are newly developed specific drugs that interfere with *P. aeruginosa* virulent gene expression without affecting bacterial growth (Carradori et al., 2020). Therefore, using a combination of QSI drugs and antibiotics is a promising strategy for treating chronic biofilm infections caused by *P. aeruginosa*. The treatment of an *in vivo P. aeruginosa* foreign-body biofilm infection with a combination of QSI drugs such as Furanone C30, ajoene, or horseradish juice extract and tobramycin showed a synergistic clearing effect on the bacteria (Christensen et al., 2012). It also has been shown that *in vitro*-grown *P. aeruginosa* biofilms in the absence of an active QS system (either by mutation of crucial QS genes or by using QSI drugs) are more susceptible to tobramycin, indicating that QSI drugs and tobramycin have combinational effects on eradicating bacterial biofilms (Høiby et al., 2010). Besides, QSIs such as patulin, penicillic acid, baicalin hydrate, and cinnamaldehyde are effective in increasing the susceptibility of *P. aeruginosa* to antibiotics (Hentzer and Givskov, 2003). These findings suggest that the synergistic treatment of *P. aeruginosa* infections is an effective strategy to prevent biofilm by first disabling the QS system by reducing bacterial virulence with QSI drugs and then killing the bacteria with antibiotics. However, there is no evidence of Pht synergistic treatment with antibiotics for *P. aeruginosa* infections. Thus, we will further examine the efficiency of Pht and its analogs in combination with antibiotics to treat *P. aeruginosa* infections.

In conclusion, using small molecules targeting the QS systems is a potential strategy for eradicating and preventing *P. aeruginosa* infections. MTb natural metabolite Pht and *P. aeruginosa* PQS are both ligands of human AhR receptors with structural similarity. In this study, Pht was confirmed as an inhibitor of the *pqs* QS system of *P. aeruginosa* with an optimal working concentration of 750 nM and is likely to compete with PQS in binding with PqsR. *Pseudomonas aeruginosa*'s key virulence factor, pyocyanin, and biofilm formation could be reduced with Pht treatment. Pht analog vitamins K_1_, K_2_, and K_3_ were also shown to inhibit the *P. aeruginosa pqs* QS system.

## Data availability statement

The original contributions presented in the study are publicly available. This data can be found in the ProteomeXchange Consortium with the dataset identifier PXD032384.

## Author contributions

TJ, LY, and GL designed the study. TJ, DL, XB, and ML performed experiments. TJ, ZC, JF, ZL, PW, XK, GZ, and AJ analyzed the results. TJ, ZC, and LY drafted and revised the manuscript. All authors contributed to the article and approved the submitted version.

## Funding

This work was supported by the National Key R&D Program of China (2021YFA1300902); Guangdong Natural Science Foundation for Distinguished Young Scholar (2020B1515020003); the Shenzhen Key Laboratory of Gene Regulation and Systems Biology, Southern University of Science and Technology (ZDSYS20200811144002008); and Shenzhen Science and Technology Program KQTD20200909113758004.

## Conflict of interest

The authors declare that the research was conducted in the absence of any commercial or financial relationships that could be construed as a potential conflict of interest.

## Publisher's note

All claims expressed in this article are solely those of the authors and do not necessarily represent those of their affiliated organizations, or those of the publisher, the editors and the reviewers. Any product that may be evaluated in this article, or claim that may be made by its manufacturer, is not guaranteed or endorsed by the publisher.
