# Supplementary material for: The AhR ligand phthiocol and vitamin K analogs as Pseudomonas aeruginosa quorum sensing inhibitors
Source: Front Microbiol. 2022 Sep 14;13:896687. doi: 10.3389/fmicb.2022.896687 (PMC9515472; doi:10.3389/fmicb.2022.896687)
Supplement: Supplementary file 1 [file Data_Sheet_1.DOCX]

**Supplementary Data**

**Supplementary Table 1** Strains and plasmids used in this study.

| **Strains or plasmids** | **Relevant genotype and/or characteristics^a^** | **Source or reference** |
| --- | --- | --- |
| **Strains:** | |  |
| PAO1 WT | ATCC *P. aeruginosa* | Lab collection |
| PAO1 P*_pqsA_*_-_*_gfp_* | Gm^a^; PAO1 containing *pqsA*-gfp (ASV) translational reporter fusion | Lab collection |
| PAO1 Δ*pqsC* | PAO1 *pqsC* mutant | Lab collection |
| PAO1 Δ*pqsC* P*_pqsA_*_-_*_gfp_* | Gm^a^; PAO1 Δ*pqsC* containing *pqsA*-gfp (ASV) translational reporter fusion | This study |
| PAO1 Δ*lasI*Δ*rhlI* | Gm^a^; PAO1 *lasI* and *rhlI* mutant | Lab collection |
| PAO1 Δ*pqsR* | PAO1 *pqsR* mutant | This study |
| **Plasmids:** | |  |
| P*_pqsA_*_-_*_gfp_* | Gm^a^ /Carb^a^; pUCP22NotI-based plasmid carrying *pqsA*-gfp (ASV) transcriptional fusion | Lab collection |
| ^a^ Description of the strains’ antibiotic resistance. Gm, gentamicin; Carb, carbenicillin resistance. | |  |


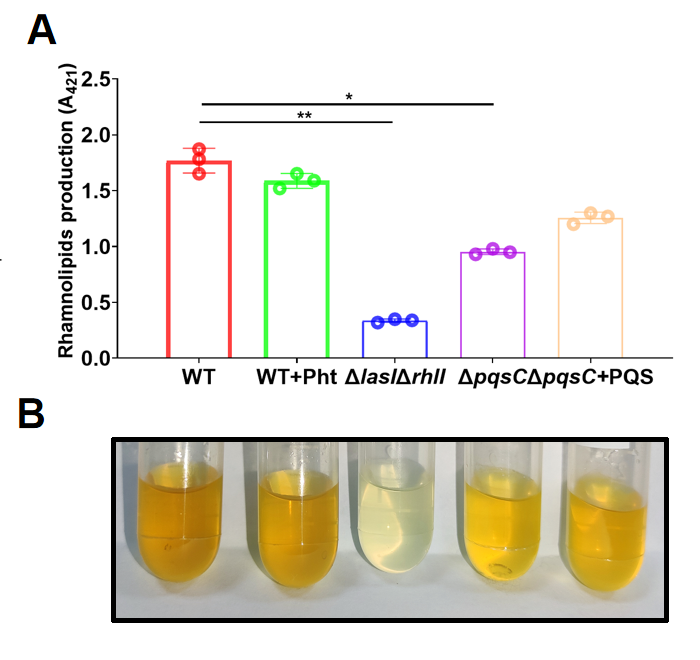


**Supplementary Figure 1.** Pht did not inhibit *P. aeruginosa* rhamnolipid productions. Data are presented as means ± SD; n = 3 independent experiments (**P* ≤ 0.05 and ***P* ≤ 0.01, Mann-Whitney *U* test).


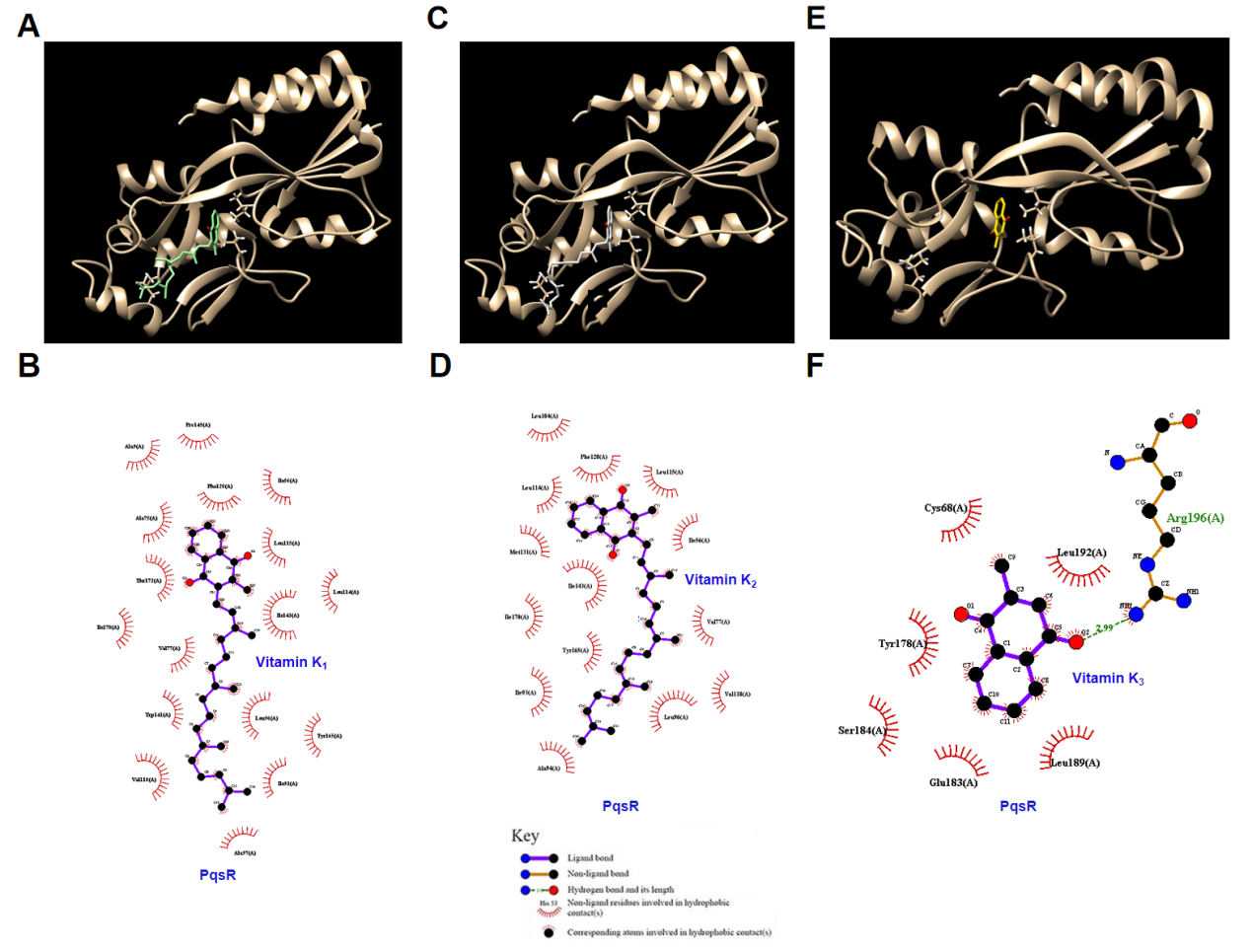


**Supplementary Figure 2.** Vitamin K_1_, K_2_, and K_3_ had the potential to bind to PqsR protein. **(A, C, E)** Interaction model of the PqsR protein with Vitamin K_1_, K_2_, and K_3_. **(B, D, F)** Interaction map between residues within the PqsR protein with Vitamin K_1_, K_2_, and K_3_.
